# Characterization of HIV-1 gp120 antibody specificities induced in anogenital secretions of RV144 vaccine recipients after late boost immunizations

**DOI:** 10.1371/journal.pone.0196397

**Published:** 2018-04-27

**Authors:** Siriwat Akapirat, Chitraporn Karnasuta, Sandhya Vasan, Supachai Rerks-Ngarm, Punnee Pitisuttithum, Sirinan Madnote, Hathairat Savadsuk, Surawach Rittiroongrad, Jiraporn Puangkaew, Sanjay Phogat, James Tartaglia, Faruk Sinangil, Mark S. de Souza, Jean-Louis Excler, Jerome H. Kim, Merlin L. Robb, Nelson L. Michael, Viseth Ngauy, Robert J. O'Connell, Nicos Karasavvas, on behalf of The RV305 Study Group

**Affiliations:** 1 Department of Retrovirology, Armed Forces Research Institute of Medical Sciences, Bangkok, Thailand; 2 The Henry M Jackson Foundation for the Advancement of Military Medicine, Bethesda, Maryland, United States of America; 3 Department of Disease Control, Ministry of Public Health, Nonthaburi, Thailand; 4 Faculty of Tropical Medicine, Mahidol University, Bangkok, Thailand; 5 Sanofi Pasteur, Swiftwater, Pennsylvania, United States of America; 6 Global Solutions for Infectious Diseases (GSID), South San Francisco, California, United States of America; 7 The Thai Red Cross AIDS Research Centre, Bangkok, Thailand; 8 US Military HIV Research Program, Walter Reed Army Institute of Research, Silver Spring, Maryland, United States of America; University of Massachusetts Medical School, UNITED STATES

## Abstract

Sexual transmission is the principal driver of the human immunodeficiency virus (HIV) pandemic. Understanding HIV vaccine-induced immune responses at mucosal surfaces can generate hypotheses regarding mechanisms of protection, and may influence vaccine development. The RV144 (ClinicalTrials.gov NCT00223080) efficacy trial showed protection against HIV infections but mucosal samples were not collected, therefore, the contribution of mucosal antibodies to preventing HIV-1 acquisition is unknown. Here, we report the generation, magnitude and persistence of antibody responses to recombinant gp120 envelope and antigens including variable one and two loop scaffold antigens (gp70V1V2) previously shown to correlate with risk in RV144. We evaluated antibody responses to gp120 A244gD and gp70V1V2 92TH023 (both CRF01_AE) and Case A2 (subtype B) in cervico-vaginal mucus (CVM), seminal plasma (SP) and rectal secretions (RS) from HIV-uninfected RV144 vaccine recipients, who were randomized to receive two late boosts of ALVAC-HIV/AIDSVAX®B/E, AIDSVAX®B/E, or ALVAC-HIV alone at 0 and 6 months. Late vaccine boosting increased IgG geometric mean titers (GMT) to gp120 A244gD in AIDSVAX®B/E and ALVAC-HIV/AIDSVAX®B/E CVM (28 and 17 fold, respectively), followed by SP and RS. IgG to gp70V1V2 92TH023 increased in AIDSVAX®B/E and ALVAC-HIV/AIDSVAX®B/E CVM (11–17 fold) and SP (2 fold) two weeks post first boost. IgG to Case A2 was only detected in AIDSVAX®B/E and ALVAC-HIV/AIDSVAX®B/E CVM. Mucosal IgG to gp120 A244gD (CVM, SP, RS), gp70V1V2 92TH023 (CVM, SP), and Case A2 (CVM) correlated with plasma IgG levels (p<0.001). Although the magnitude of IgG responses declined after boosting, anti-gp120 A244gD IgG responses in CVM persisted for 12 months post final vaccination. Further studies in localization, persistence and magnitude of envelope specific antibodies (IgG and dimeric IgA) in anogenital secretions will help determine their role in preventing mucosal HIV acquisition.

## Introduction

Despite numerous recent advances in HIV prevention, a preventive vaccine remains a high priority to prevent ongoing transmission of the virus [1]. Sexual acquisition occurs across the genital and rectal mucosa surfaces following sexual activity with HIV-infected partners [2]. In humans, innate mucosal immune factors have been associated with protection from HIV infection, and mucosal HIV-specific antibodies exhibit viral neutralization and/or inhibition of HIV infection [3–6]. Concentrations of IgG in cervico-vaginal mucus (CVM) and semen are higher than IgA, whereas rectal secretions (RS) contain higher levels of secretory IgA than IgG [7–9]. Furthermore, half of the antibodies in female genital secretions are produced systemically by plasma cells in peripheral blood and transported, while more than 90% of intestinal antibodies are locally produced in the lamina propria [10]. Studies in animal models have demonstrated the successful generation of mucosal HIV-specific antibodies by immunizing with HIV antigens either systemically, mucosally, or a combination of both routes [11–13].

The RV144 trial of a prime-boost vaccine regimen consisting of recombinant canarypox priming immunogen, ALVAC-HIV (vCP1521), and bivalent AIDSVAX®B/E glycoprotein (gp)120 (MN and A244) protein boosting demonstrated a modest 31.2% protective efficacy against HIV infection at 42 months of follow-up; however, vaccine efficacy was 60.5% at 12 months after initial vaccination [14, 15]. Post hoc analysis revealed two factors correlated to HIV infection risk: plasma IgG binding antibodies to variable loops 1 and 2 (V1V2) of gp120 envelope (Env) protein inversely correlated with HIV infection risk among RV144 vaccine recipients, while plasma monomeric IgA binding antibodies to viral Envs directly correlated with risk [16–19]. Additionally, a sieve analysis identified vaccine-associated genetic signatures in the V2 region, further providing evidence that vaccination-induced immune responses directed against the V2 loop were associated with protection conferred by the RV144 regimen [20]. However, HIV-specific immune responses in anogenital secretions were not characterized because mucosal specimens were not collected in RV144.

In the current study, RV305 (ClinicalTrials.gov NCT01435135), we report the presence of HIV-specific antibodies in anogenital secretions after intramuscular immunizations with RV144 vaccine components. One hundred and sixty two HIV-uninfected RV144 vaccine recipients were randomized to receive two late boosts of either a combination of ALVAC-HIV/AIDSVAX®B/E or AIDSVAX®B/E alone or ALVAC-HIV alone [21]. Antibody responses in anogenital secretions were collected and assessed at five different time points throughout the study. We demonstrate a strong correlation of antibody responses in anogenital secretions with those in plasma. Our results provide evidence that intramuscular immunizations induced anogenital IgG to gp120 that might have contributed to prevention of HIV infection.

## Materials and methods

### Study design

**Fig 1:**
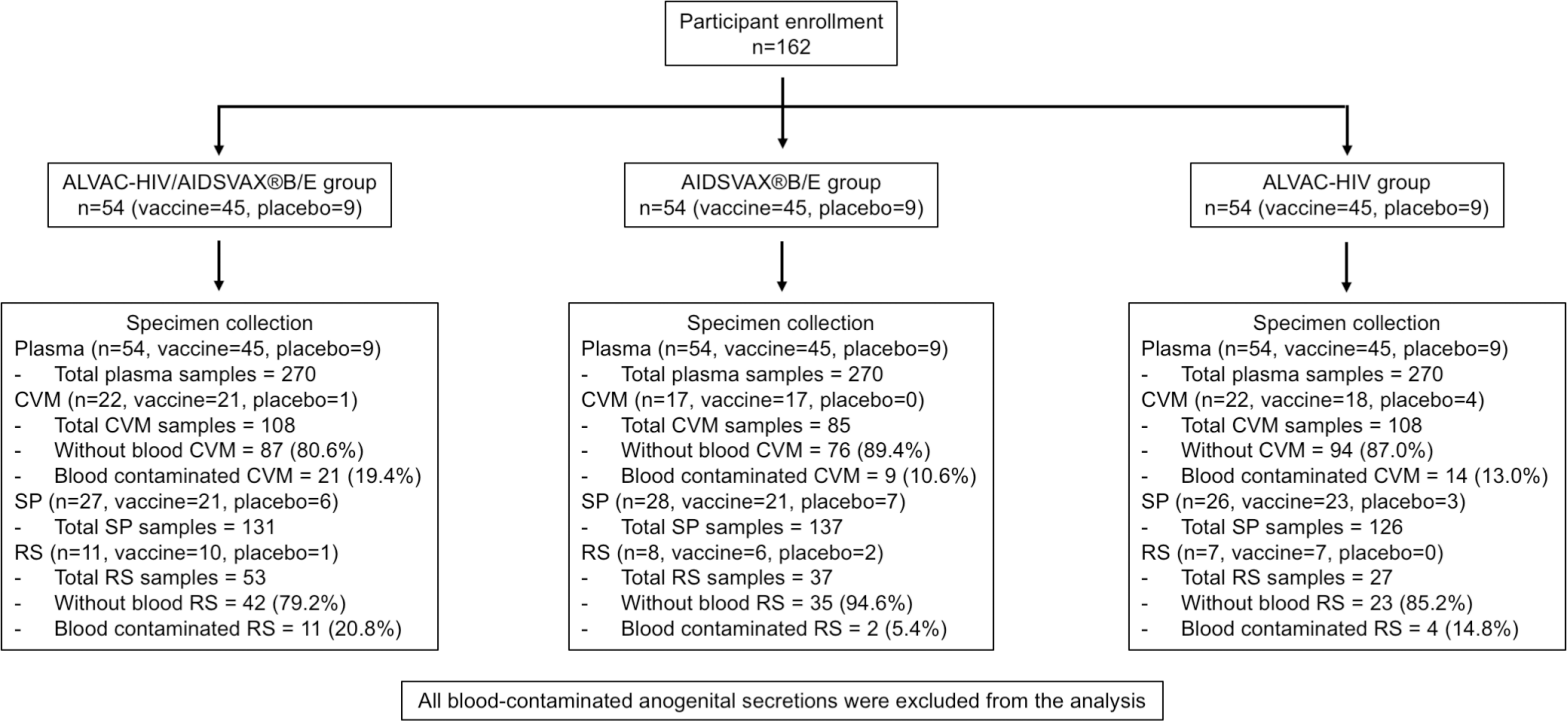
Specimen collection algorithm. Blood specimens were collected from each study participant to obtain plasma. Cervico-vaginal mucus (CVM) was collected from consenting female participants. Seminal plasma (SP) and rectal secretions (RS) were collected from consenting male participants. Blood contamination was tested on both CVM and RS using Hemoccult® SENSA® test kit (Beckmann Coulter, Brea, CA). All blood contaminated specimens were excluded from the analysis. Blood contamination was not tested on SP.

In the RV305 clinical trial, 162 healthy HIV-uninfected vaccine recipients who completed all vaccination series from RV144 [14] 6.0 to 8.3 years earlier (mean = 7.2 years since last RV144 vaccination) were randomized into three groups, each composed of 45 vaccine and 9 placebo recipients, to receive two intramuscular immunizations at 0 and 6 months of one of three booster options or placebo products. Group 1 received ALVAC-HIV/AIDSVAX®B/E or ALVAC/AIDSVAX placebo, Group 2 received AIDSVAX®B/E or AIDSVAX placebo, and Group 3 received ALVAC-HIV or ALVAC placebo (Fig 1) [21]. The protocol received approval from ethics committees and institutional review boards of the Walter Reed Army Institute of Research, the Thai Ministry of Public Health, the Royal Thai Army Medical Department, the Faculty of Tropical Medicine, Mahidol University, the Faculty of Medicine, Chulalongkorn University, and Siriraj Hospital. Written informed consent was obtained from all participants.

### Clinical specimen collection, processing and storage

Venous blood, CVM, seminal plasma (SP) and RS were collected and tested at study entry (week 0), 2 weeks post first (week 2) and second boosts (week 26), and weeks 48 and 72. All participants were HIV-uninfected throughout the study.

Venous blood collected in Acid Citrate Dextrose (ACD) tubes from each participant were centrifuged at 800 g for 15 minutes to obtain plasma.

Optional CVM was collected from 61 consenting female participants (Group 1: 21 vaccine and 1 placebo recipients, Group 2: 17 vaccine recipients, and Group 3: 18 vaccine and 4 placebo recipients) (Fig 1) using Instead Softcup™ (Evofem, San Diego, CA). Participants were asked to refrain from sexual activities for at least 72 hours prior to CVM collection. Wherever possible, collections were timed to avoid menses. Softcups were inserted and removed by participants and time of intravaginal retention was recorded ranging from 4.02 to 12.35 hours. Secretions were processed by placing Softcups in 50 mL conical tubes and centrifuged at 500 g for 10 minutes at +4°C. To ensure that antibodies in secretions were not derived from blood contamination, CVM was tested for the presence of blood using Hemoccult^®^ SENSA^®^ test kit (Beckmann Coulter, Brea, CA). When possible, the participant was asked to provide another sample when blood was detected in CVM. Secretions were suspended in two volumes of phosphate-buffered saline (PBS) (Life Technologies, Grand Island, NY) containing 1X Calbiochem protease inhibitor cocktail set I (EMD Millipore Corp., Billerica, MA) to prevent antibody proteolysis.

SP was isolated from semen collected from 81 male participants (Group 1: 21 vaccine and 6 placebo recipients, Group 2: 21 vaccine and 7 placebo recipients, and Group 3: 23 vaccine and 3 placebo recipients) (Fig 1) by masturbation without lubricants. Participants were asked to refrain from sexual activities for at least 72 hours prior to semen donation and specimens were kept at 2–6°C during transport for processing. Specimens were kept for at least one hour at 4°C± 2°C prior to processing to liquefy semen. Semen was diluted 1:1 with PBS (Life Technologies, Grand Island, NY), mixed and centrifuged for 10 minutes (1,200 g) to separate cells from SP. Supernatant was mixed with 10% extraction solution containing protease inhibitor cocktail tablets (cOmplete™ ULTRA Tablets, Mini, Roche Diagnostics, Mannheim, Germany).

RS were collected from 26 male participants (Group 1: 10 vaccine and 1 placebo recipients, Group 2: 6 vaccine and 2 placebo recipients, and Group 3: 7 vaccine recipients) (Fig 1) by placing Merocel^®^ Schindler ear packing sponges (Medtronic Xomed, Jacksonville, FL) anterior to the dentate line for 2–5 minutes, avoiding sponge placement in locations containing fecal matter. Sponges were weighed before and after collection to determine sample weight. RS were extracted three times with 0.8mL of PBS (Life Technologies, Grand Island, NY) containing Calbiochem protease inhibitor cocktail set I (EMD Millipore Corp., Billerica, MA) and centrifuged at 2,300 g for 5 minutes at +4°C. Supernatant was tested for blood contamination using Hemoccult^®^ SENSA^®^ test kit (Beckmann Coulter, Brea, CA), and pooled for each participant.

All plasma and anogenital specimens were aliquoted and stored at <-80°C until testing.

### Recombinant proteins

Recombinant gp120 HIV-1 CRF01_AE A244gD protein (A244gD) with identical amino acid sequence to the one used in RV144 was purified using *Galanthous nivalis* lectin columns as described previously [22]. Subtype B Case A2 (Case A2) and CRF01_AE 92TH023 (92TH023) gp70V1V2 scaffold proteins were synthesized as previously described [23].

### Antibody binding ELISA assays

IgG and IgA antibody responses to A244gD protein [17], and gp70V1V2 92TH023 and Case A2 [18, 24, 25] were assessed by ELISA as previously described [17]. Briefly, ELISA for all proteins was performed in 96-well U-bottom Immulon 2HB plates (Thermos Scientific, Rochester, NY) coated with 1 μg/mL of proteins in D-PBS (Sigma-Aldrich, Saint Louis, MO) at 4°C overnight. Plates were washed and serial two-fold dilutions of all secretions were added to wells with initial dilutions of CVM and SP of 1:100 (gp120 A244gD) and 1:25 for gp70V1V2 scaffolds. The initial dilution for RS was 1:5 for all antigens. Plates were incubated at room temperature for 2 hours. Plates were washed and color was developed with horseradish peroxidase (HRP) conjugated either to goat anti-human IgG or goat anti-human IgA (Bethyl Laboratories, Montgomery, TX) at 1:25,000 dilution and ABTS ELISA HRP substrate (KPL, Gaithersburg, MD). Plates were read at an absorbance of A405 nm (Spectramax 340 PC ELISA reader, Molecular Devices, Downingtown, PA). Human reference serum (Bethyl Laboratories, Montgomery, TX) was used as a positive control.

Total IgG and IgA antibody specific to goat anti-human IgG-Fc antibody (Bethyl Laboratories, Montgomery, TX) and goat anti-human IgA antibody (Bethyl Laboratories, Montgomery, TX) were assessed on all anogenital secretions following above ELISA procedure.

### Statistical analysis

Data analysis and graphs were generated using GraphPad Prism version 7.01 for Windows (GraphPad Software, La Jolla, CA). Titers were expressed as the reciprocal of the highest dilutions that yielded an absorbance value of A405 nm greater than 0.25 (2.5-fold the absorbance of wells without capture antigen). The positive reciprocal titers were above the cut-off level (0.5-fold the reciprocal titers of the initial dilution of specimens). Descriptive results are presented as medians with minimum and maximum ranges. Antibody concentration results are presented as geometric mean titers (GMT) which were calculated with associated 95% confidence intervals. Statistical comparisons were assessed using non-parametric Mann-Whitney U tests. Correlation of antibody responses was assessed using Spearman’s Rank Correlation Coefficient test. A 2-sided p-value of <0.05 was considered significant.

## Results

### Determination of total IgG and IgA concentrations in anogenital secretions

Total IgG and IgA concentrations in anogenital secretions were measured at study entry, and 2 weeks post first and second boosts (Table 1). Median (range) concentrations of total IgG in CVM (602.6 (68.3–5,037.0) μg/mL) and SP (44.5 (11.5–154.1) μg/mL) were approximately two-fold higher than those of total IgA (376.4 (7.1–1,576) and 22.1 (1.4–221.5) μg/mL, respectively). Conversely, total IgG (29.5 (1.8–484.8) μg/mL) in RS was approximately five-fold lower than total IgA (142.2 (2.0–3,677.0) μg/mL). Of 301 CVM and 117 RS samples collected, 44 (14.6%) and 17 (14.5%) were positive for blood contamination, respectively (Fig 1). Although intravaginal retention time of Instead SoftCup ranged from 4.02–12.35 hours (median hours = 6.07), it did not correlate with yield of collected CVM (p = 0.283).

**Table 1:** Concentration of total IgG and IgA in anogenital secretions that did not contain blood.

	Cervico-vaginal mucus (CVM)	Seminal plasma (SP)	Rectal secretions (RS)
No. of collected specimens	301	394	117
Median (range) total IgG (μg/mL)	602.6 (68.3–5,037.0), n = 156	44.5 (11.5–154.1), n = 238	29.5 (1.8–484.8), n = 65
Median (range) total IgA (μg/mL)	376.4 (7.1–1,576.0), n = 156	22.1 (1.4–221.5), n = 238	142.2 (2.0–3,677.0), n = 65

Total IgG and IgA in samples without blood and blood-contaminated samples were compared to determine if the presence of blood increased the concentration of antibodies in secretions. We observed significantly higher concentrations of IgG in blood-contaminated CVM (p = 0.018) and RS (p = 0.022) and of IgA in blood-contaminated CVM only (p = 0.048). Although there was a trend in RS for higher IgA in blood containing samples, significance was not reached (p = 0.431). However, the number of matched RS samples used for the analysis was small (N = 5–7). We excluded all blood-contaminated samples from analysis to avoid inclusion of antibody responses normally found in blood. SP samples were not tested for blood contamination.

### HIV-specific antibody responses in anogenital secretions

**Fig 2:**
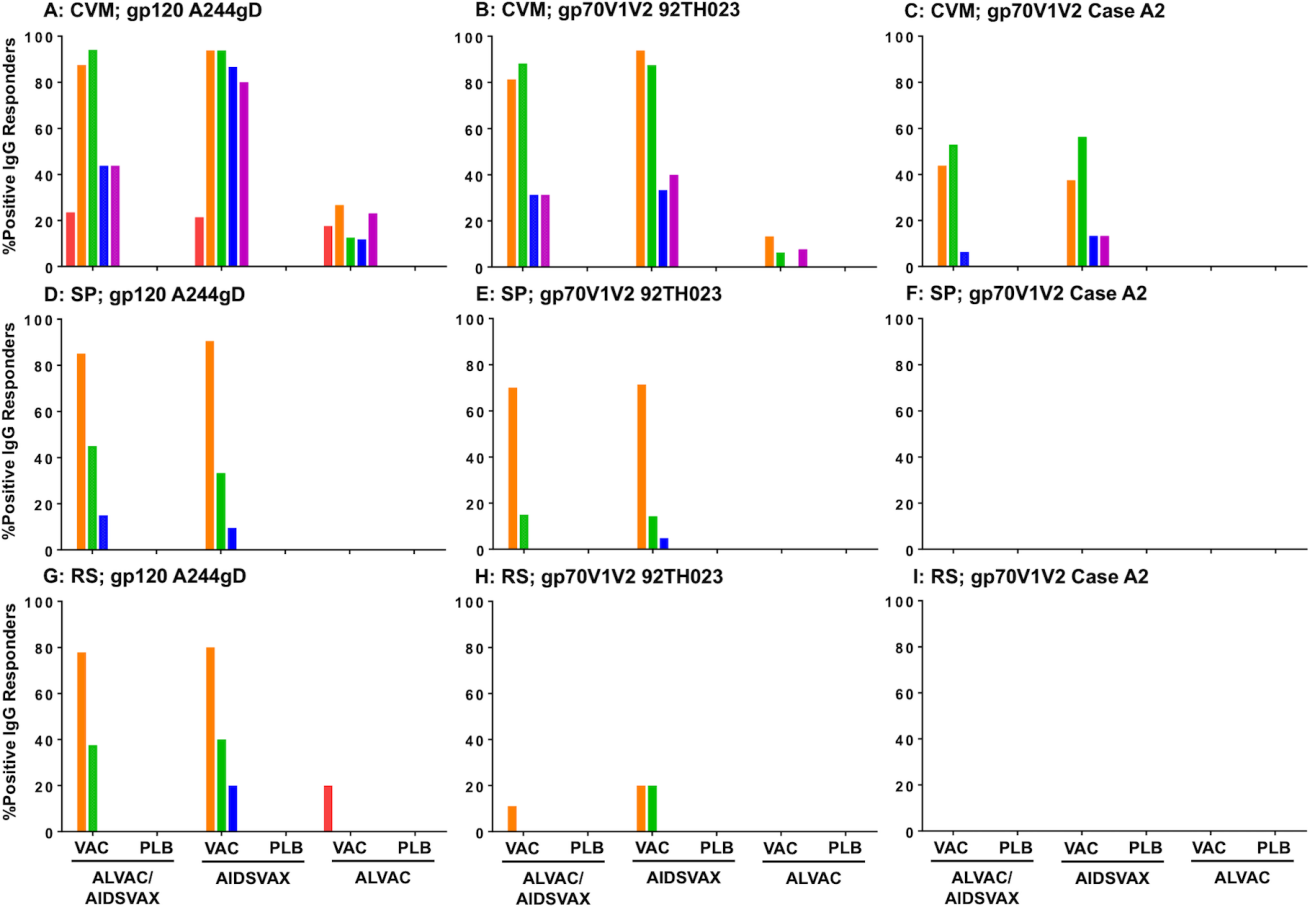
Percent of positive IgG responders to all HIV antigen tested in anogenital secretions. Percent of positive IgG responders to gp120 A244gD (CRF01_AE), gp70V1V2 92TH023 (CRF01_AE) and gp70V1V2 Case A2 (subtype B) in CVM (A-C), SP (D-F), and RS (G-I) are shown. Each time point is color coded; red, week 0; orange, week 2; green, week 26; blue, week 48; magenta, week 72. CVM and RS were not collected from placebo recipient of groups AIDSVAX^®^B/E and ALVAC-HIV, respectively, at any time point. VAC = vaccine recipients; PLB = placebo recipients; ALVAC/AIDSVAX = ALVAC-HIV/AIDSVAX^®^B/E group; AIDSVAX = AIDSVAX^®^B/E group; ALVAC = ALVAC-HIV group.

HIV-specific IgG antibody responses were not detected in any secretions from placebo recipients at baseline (Fig 2), which could be due to the low number of samples collected (1–7 samples). IgA responses to all HIV antigen tested were not detected in any secretions of both vaccine and placebo recipients.

### IgG antibody responses to gp120 A244gD protein and gp70V1V2 scaffolds in CVM

**Fig 3:**
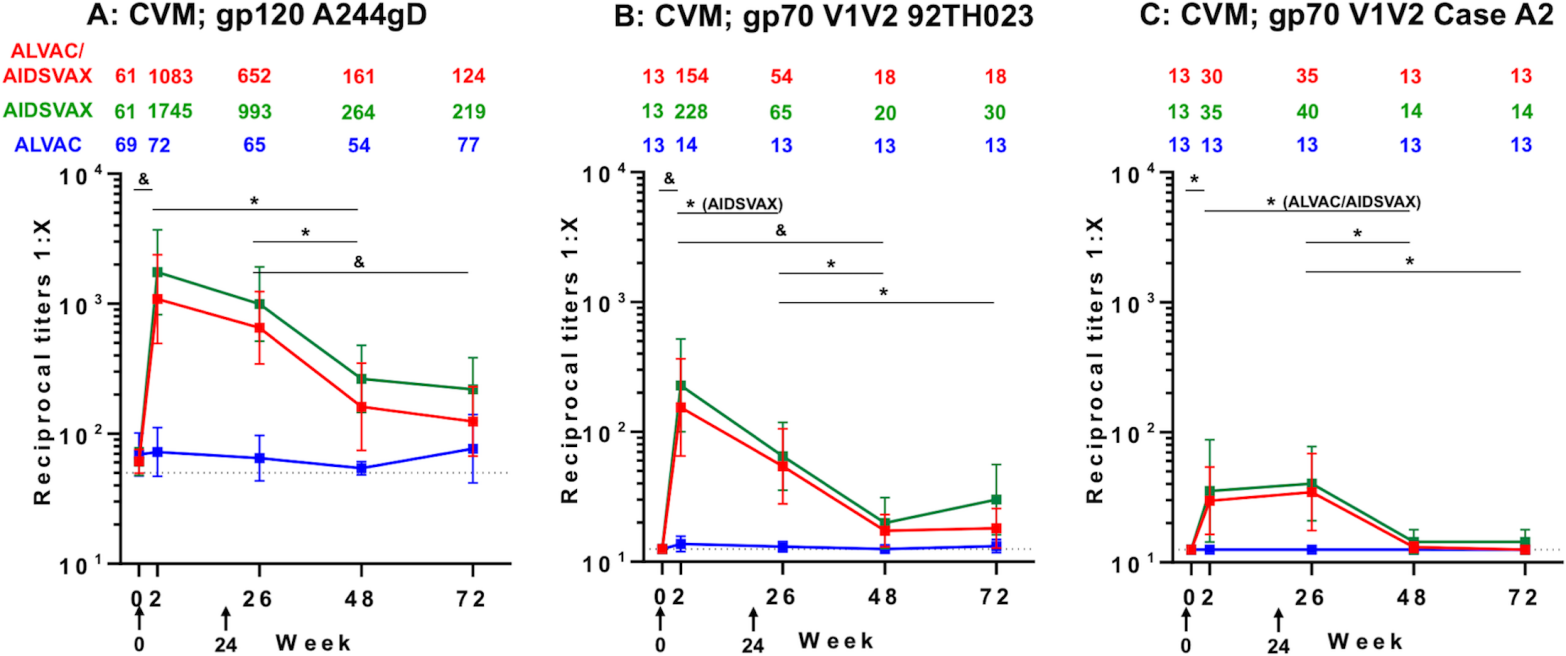
IgG binding antibody responses to gp120 A244gD and gp70V1V2 scaffolds in cervico-vaginal mucus (CVM). Reciprocal titers of IgG binding antibody responses to (A) gp120 A244gD (CRF01_AE), (B) gp70V1V2 92TH023 (CRF01_AE) and (C) gp70V1V2 Case A2 (subtype B) in CVM are shown along with numeric depiction of geometric mean titers above panels. Each group is color coded; red, ALVAC-HIV/AIDSVAX^®^B/E (ALVAC/AIDSVAX); green, AIDSVAX^®^B/E (AIDSVAX); blue, ALVAC-HIV (ALVAC). Error bars depict 95% confidence intervals. The cut-off level of responses (0.5-fold the reciprocal titers of initial dilution of specimens) is shown by the dotted line. RV305 vaccine administration time points are indicated by black arrows (weeks 0 and 24). The non-parametric Mann-Whitney U Test was used to assess within-group comparison of IgG responses between time points indicated by horizontal black bars. Comparisons reaching statistical significance at the level of p<0.05 are shown. *p<0.05 to 0.001, ^&^p<0.001.

Responses to gp120 A244gD were measured in ALVAC-HIV/AIDSVAX^®^B/E (23.5%, 4/17), AIDSVAX^®^B/E (21.4%, 3/14) and ALVAC-HIV (17.6%, 3/17) groups at week 0 (GMT: 61–69) (Fig 2). ALVAC-HIV immunization did not increase HIV-specific antibody levels relative to baseline responses (p = 0.657; Fig 3A). ALVAC-HIV/AIDSVAX^®^B/E and AIDSVAX^®^B/E vaccinations induced significant increases in antibody levels that were similar in magnitude. Two weeks post first immunization (week 2), antibody responses to gp120 A244gD increased significantly from baseline (p<0.001) in both ALVAC-HIV/AIDSVAX^®^B/E (GMT: 1,083) and AIDSVAX^®^B/E (GMT: 1,745) groups. However, an additional boost did not increase the magnitude of the response (GMT: 652 and 993, respectively) (Fig 3A). Although antibody responses after the second boost were lower than post first boost, the magnitude was not significant (p = 0.114 and 0.206, respectively). At week 48, GMT declined significantly in the ALVAC-HIV/AIDSVAX^®^B/E (161, p = 0.011) and AIDSVAX^®^B/E (264, p = 0.002) groups. GMT remained stable and approximately the same at week 72, GMT = 124 (p = 0.643) and 219 (p = 0.744), respectively. In general, positive responders at week 48 remained above cutoff at week 72.

CVM IgG responses to gp70V1V2 92TH023 and Case A2 scaffolds in all vaccination groups were at baseline level (GMT = 13) at study entry (Fig 3B and 3C). Following the first immunization, antibody responses to gp70V1V2 92TH023 were induced in both the ALVAC-HIV/AIDSVAX^®^B/E (GMT: 154, p<0.001) and AIDSVAX^®^B/E (GMT: 228, p<0.001) groups, but the responses were not significantly different between groups (p = 0.673, Fig 3B). As seen with gp120, immunizations in the ALVAC-HIV group induced responses similar to study entry and significantly lower than those in the other two groups (p<0.02). Additional boosting with AIDSVAX^®^B/E or ALVAC-HIV/AIDSVAX^®^B/E did not further increase V1V2-specific responses (GMT: 65 (p = 0.015) and 54 (p = 0.069), respectively). Antibody levels decreased significantly but remained above baseline levels (week 0) in both groups at weeks 48 (GMT: 20 and 18; p<0.005) and 72 (GMT: 30 and 18; p<0.05).

CVM antibody responses to gp70V1V2 Case A2 scaffold were lower than those to gp70V1V2 92TH023. Following the first boost, GMT of ALVAC-HIV/AIDSVAX^®^B/E and AIDSVAX^®^B/E groups increased similarly from 13 and 13 to 30 and 35, respectively, and the responses remained approximately the same after the second boost (GMT: 35 and 40) (Fig 3C). In both ALVAC-HIV/AIDSVAX^®^B/E and AIDSVAX^®^B/E groups, the responses declined significantly and were close to study baseline levels at weeks 48 (GMT: 13 and 14; p>0.48) and 72 (GMT: 13 and 14; p>0.48).

### IgG antibody responses to gp120 A244gD protein and gp70V1V2 scaffolds in SP

**Fig 4:**
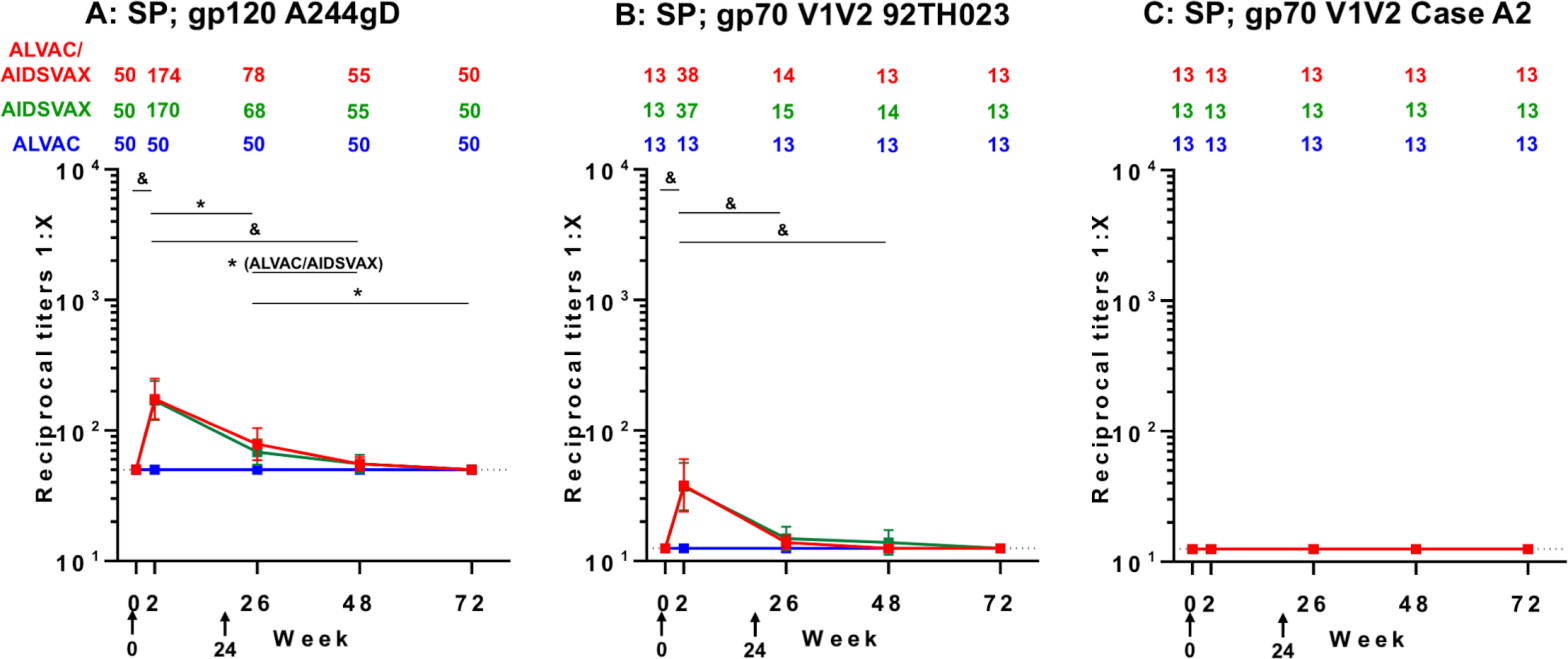
IgG binding antibody responses to gp120 A244gD and gp70V1V2 scaffolds in seminal plasma (SP). Reciprocal titers of IgG binding antibody responses to (A) gp120 A244gD (CRF01_AE), (B) gp70V1V2 92TH023 (CRF01_AE) and (C) gp70V1V2 Case A2 (subtype B) in SP are shown along with numeric depiction of geometric mean titers above panels. Each group is color coded; red, ALVAC-HIV/AIDSVAX^®^B/E (ALVAC/AIDSVAX); green, AIDSVAX^®^B/E (AIDSVAX); blue, ALVAC-HIV (ALVAC). Error bars depict 95% confidence intervals. The cut-off level of responses (0.5-fold the reciprocal titers of the initial dilution of specimens) is shown by the dotted line. RV305 vaccine administration time points are indicated by black arrows (weeks 0 and 24). The non-parametric Mann-Whitney U Test was used to assess within-group comparison of IgG responses between time points indicated by black bars. Comparisons reaching statistical significance at the level of p<0.05 are shown. *p<0.05 to 0.001, ^&^p<0.001.

Prior to immunization, IgG antibody responses in SP to all antigens were at baseline level (gp120 A244gD, GMT = 50; gp70V1V2 92TH023 and Case A2, GMT = 13). Boosting immunizations did not induce the responses in the ALVAC-HIV group for all HIV antigens tested (Fig 4A–4C). IgG responses to gp120 A244gD were detected after the first vaccination in both ALVAC-HIV/AIDSVAX^®^B/E and AIDSVAX^®^B/E groups with GMT = 174 and 170, respectively (Fig 4A). After the second boost, GMT did not increase further and were lower than post first boost, 78 (p = 0.001) and 68 (p<0.001), respectively. Antibody responses declined in both groups at weeks 48 (GMT: 55 and 50; p = 0.04 and 0.118, respectively) and 72 reaching baseline levels (GMT: 50 and 50; p = 0.231 and 0.487, respectively) (Fig 4A).

IgG responses to gp70V1V2 92TH023 scaffold were also detected in SP after the first vaccination with ALVAC-HIV/AIDSVAX^®^B/E and AIDSVAX®B/E groups (GMT: 38 and 37; p<0.001) (Fig 4B). However, after the second boost, the responses did not increase and were similar to study baseline (GMT: 14 and 15; p = 0.107). Antibodies in both groups were identical at week 72 to those at baseline. IgG responses to gp70V1V2 subtype B (Case A2) in SP were undetectable in all groups (Fig 4C).

### IgG antibody responses to gp120 A244gD protein and gp70V1V2 scaffolds in RS

**Fig 5:**
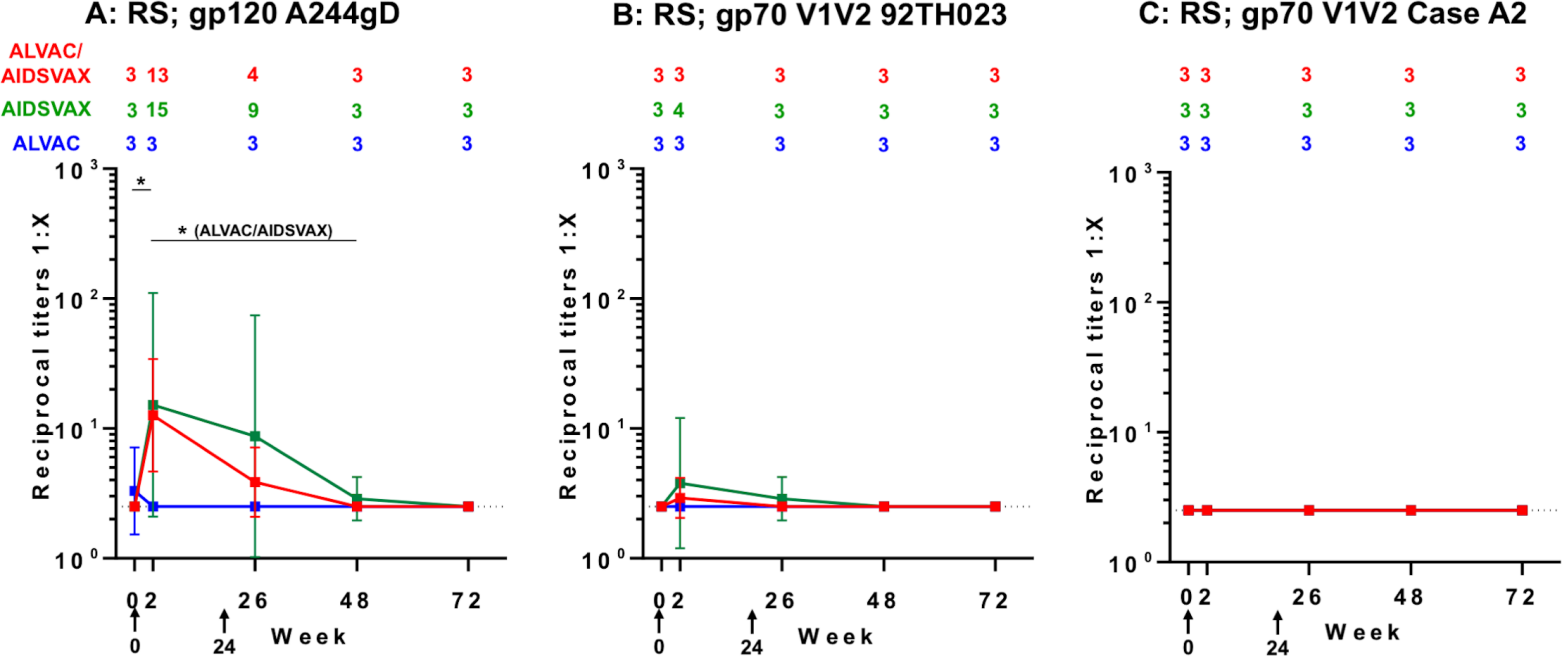
IgG binding antibody responses to gp120 A244gD and gp70V1V2 scaffolds in rectal secretions (RS). Reciprocal titers of IgG binding antibody responses to (A) gp120 A244gD (CRF01_AE), (B) gp70V1V2 92TH023 (CRF01_AE) and (C) gp70V1V2 Case A2 (subtype B) in RS are shown along with numeric depiction of geometric mean titers above panels. Each group is color coded; red, ALVAC-HIV/AIDSVAX^®^B/E (ALVAC/AIDSVAX); green, AIDSVAX^®^B/E (AIDSVAX); blue, ALVAC-HIV (ALVAC). Error bars depict 95% confidence intervals. The cut-off level of responses (0.5-fold the reciprocal titers of the initial dilution of specimens) is shown by the dotted line. RV305 vaccine administration time points are indicated by black arrows (weeks 0 and 24). The non-parametric Mann-Whitney U Test was used to assess within-group comparison of IgG responses between time points indicated by black bars. Comparisons reaching statistical significance at the level of p<0.05 are shown. *p<0.05 to 0.001, ^&^p<0.001.

At baseline, IgG to gp120 A244gD was detected in one RS sample in the ALVAC-HIV group but vaccinations in this group did not induce antibody responses (Fig 5A–5C). Following the first boost (week 2), IgG responses to gp120 A244gD were detected in ALVAC-HIV/AIDSVAX^®^B/E and AIDSVAX^®^B/E groups (GMT: 13 and 15; p = 0.002 and 0.015, respectively) (Fig 5A). A second boost did not increase these responses further. Rectal antibody responses declined rapidly and were undetected in all groups at week 72. Low IgG responses to gp70V1V2 92TH023 were detected in RS of ALVAC-HIV/AIDSVAX^®^B/E group at week 2 and AIDSVAX^®^B/E group at weeks 2 and 26 (Fig 5B). These responses were transient and declined to baseline at week 48. IgG responses to gp70V1V2 Case A2 were undetectable in RS (Fig 5C).

### Correlation of IgG responses between plasma and anogenital secretions

**Fig 6:**
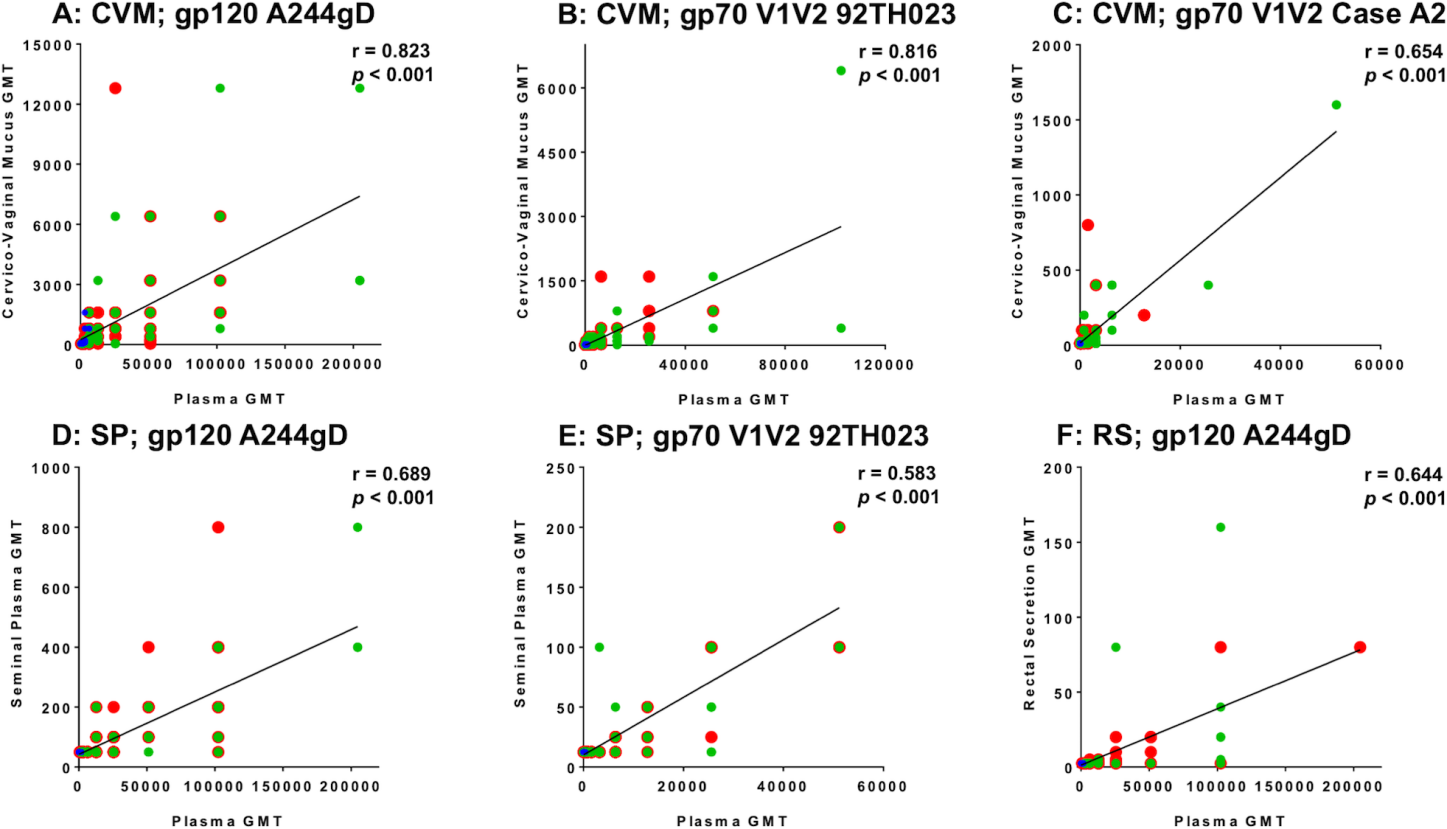
Correlation of IgG responses in anogenital secretions and plasma. Spearman’s rank correlations of IgG responses for gp120 A244gD (CRF01_AE), gp70V1V2 92TH023 (CRF01_AE) and gp70V1 V2 Case A2 (subtype B) at weeks 2, 26, 48 and 72 in matched CVM (A-C), SP (D-E) and RS (F), and plasma of RV305 vaccine recipients are shown. Each group is color coded; red, ALVAC-HIV/AIDSVAX^®^B/E; green, AIDSVAX^®^B/E; blue, ALVAC-HIV. Numeric values above each plot depict r- and p-values. Significant p-value <0.05, GMT = Geometric Mean Titer.

Anogenital secretion IgG responses to HIV proteins tested showed similar patterns to plasma responses [21]. We therefore, investigated the correlation of IgG responses in plasma and all anogenital secretions collected at different time points after the first immunization until the end of study (weeks 2, 26, 48 and 72). HIV-specific IgG titers in CVM showed a significant positive correlation with those in plasma (p<0.001) for gp120 A244gD and both scaffolds, (Fig 6A–6C). A significant correlation of IgG antibody responses for gp120 A244gD and gp70V1V2 92TH023 was also found between SP and plasma (p<0.001) (Fig 6D and 6E). In RS, only IgG responses to gp120 A244gD had a significant positive correlation with plasma levels (p<0.001) (Fig 6F).

## Discussion

Mucosal surfaces are a major route for HIV infection but the precise mechanisms by which the virus penetrates mucosal barriers to establish infection are not completely understood. Similarly, because no highly efficacious HIV vaccine has been developed, vaccinologists are considering multiple potential mechanisms of protection including neutralization and non-neutralizing functions [26, 27]. However, in order for vaccine-induced antibody-mediated protection to occur at the mucosal surfaces, antibodies must be present at the time of viral encounter. This study demonstrates the induction of HIV-specific antibodies by using immunogens of the RV144 regimen as late boosts 6–8 years following RV144 immunization, including responses to antigens that inversely correlated with risk in the RV144 trial [28].

Collection of vaginal and rectal secretions for the characterization of antibodies in vaccine and natural infections has been technically challenging because samples collected using lavage introduce dilution factors that were difficult to determine [29, 30]. In RV305, we used Merocel^®^ sponges and Instead Softcup™ to collect undiluted RS and CVM, respectively, thereby eliminating dilution factor differences. With regard to total immunoglobulins, in CVM and SP, IgG was predominant with a mean concentration twice that of IgA, although the latter was the predominant isotype in RS. Higher concentrations of IgG in CVM and SP, and higher IgA concentrations in RS have been reported previously [7–10].

Low levels of residual antibody responses to gp120 A244gD were detected in CVM at week 0. It was reported previously that immunoglobulins in female genital secretions originate from either local production or antibody transport from blood circulation to mucosal compartments [10]. Following immunization, we detected IgG Env-specific antibodies in all secretions except the ALVAC-HIV alone where they were either very weak (CVM, gp120 antigen only) or undetectable (SP and RS). Antibody responses in the ALVAC-HIV/AIDSVAX^®^B/E and AIDSVAX^®^B/E groups were indistinguishable, suggesting that the AIDSVAX^®^B/E proteins were necessary in driving the antibody response. It remains to be determined whether the presence of ALVAC-HIV impacts the quality of antibodies generated in anogenital secretions. Overall, antibody levels in CVM and SP in the ALVAC-HIV/AIDSVAX^®^B/E and AIDSVAX^®^B/E groups two weeks post first immunization were higher than those after the second immunization, as previously reported for plasma [21], whereas in RS, responses were similar between the first and second immunizations. Notably, IgG to gp70V1V2 Case A2, which correlated inversely to risk of HIV infection in RV144 [28], was only detected in CVM but not in SP and RS. This difference could be related to the detection sensitivity, levels and quality of antibodies in these samples. Antibody levels in both SP and RS were transient and dropped to baseline levels at week 48. In CVM, antibody titers declined significantly at weeks 48 and 72 after the second immunization but remained above baseline indicating that HIV-specific responses were more persistent in this compartment. Naturally, CVM contains higher concentrations of IgG than SP and RS [31] providing an explanation for the higher concentrations of antigen-specific antibodies detected in CVM.

CVM contained the highest HIV-specific antibodies compared to SP and RS. Antibody titers to gp120 A244gD were 6–14 fold higher than SP, and 83–163 fold higher than RS. Similarly, CVM titers to gp70V1V2 92TH023 were 4–6 fold higher than SP, and 20–60 fold higher than RS. Although the level and specificity of antibodies in mucosal secretions that are needed to provide protection are unknown, higher levels of HIV-specific IgG antibodies in CVM may provide better protection via this route of infection. Though intrarectal HIV transmission could be due to microtrauma during sexual intercourse leading to direct inoculation of HIV in the blood [32], vaccine development that generates higher levels of HIV-specific IgG and/or secretory IgA antibodies in RS may provide additional protection.

Antibody concentrations in mucosal secretions positively correlated with those in plasma, suggesting transudation of antibodies between systemic and mucosal compartments. It has been reported previously that antibodies in female genital mucosa and intestine are derived from the systemic circulation [10]. Neonatal Fc receptors (FcRn) on intestinal and genital epithelium were shown to mediate trans-epithelial transport of IgG into the lumen of intestinal and genital tracts where IgG could acquire antigens and form immune complexes [33–35]. Macrophages and monocytes expressing FcRs for IgG and IgA abundantly below human vaginal epithelium might also mediate both IgG and IgA effector functions [36]. In CVM, there was a positive correlation for gp120 A244gD and gp70V1V2 scaffold proteins. In SP, correlation was detected for gp120 A244gD and to gp70V1V2 92TH023 but no correlation was found for Case A2 scaffold. In RS, positive correlation was only observed for gp120 A244gD but not the scaffold proteins. This is likely due to the low number of responders and magnitude of antibody response (limit of detection of weak signals by ELISA) in RS. Taken together, correlation of antibody titers in plasma and mucosal secretions indicates that systemic antibodies likely contributed to the levels of IgG gp120-specific antibodies detected in anogenital secretions.

Monomeric IgA is predominantly found in plasma, whereas either dimeric or polymeric IgA is found in mucosal compartments [37]. Monomeric IgA to gp120 has been detected in plasma from RV144 vaccine recipients [38]. However, monomeric IgA cannot be transported by the polymeric immunoglobulin receptor (pIgR), which only transports dimeric IgA (secretory IgA) [39]. Though CVM and SP contain monomeric IgA generated by local production, it cannot be transported from plasma to secretions explaining lack of IgA to gp120 in CVM and SP following intramuscular administration with this vaccine. Dimeric IgA is the predominant IgA form in RS [10] but we did not detect dimeric IgA to any antigen we tested. Anogenital secretions were not collected as part of the RV144 trial, so dimeric IgA levels in mucosal specimens could not be evaluated. Naturally low IgG concentrations in RS and rapid decline of antibodies to immunization antigens may pose a challenge in generating robust IgG responses to HIV in the rectum. Developing vaccine regimens that induce dimeric IgA to HIV antigens in secretions may increase protection of HIV-1 transmissions via the mucosal routes [40].

Preclinical trials in animal models revealed that mucosal immunization with HIV antigens could induce both systemic and mucosal protective antibodies [41–45]. Other studies demonstrated the generation of mucosal immune responses and protective anti-HIV antibodies by systemic immunization and/or mucosal administration of HIV immunogens. Intramuscular immunized macaques were completely protected from intra-rectal challenge with simian-human immunodeficiency virus (SHIV) when vaccinated with a vaccine regimen consisting of alphavirus replicon particles (prime) and trimeric envelope glycoprotein boosts. Mucosal HIV-specific IgG or IgA was not detected in rectal or vaginal secretions [11]. Dimeric IgAs delivered directly into the rectal lumen have been reported to prevent SHIV acquisition [46]. Another study demonstrated induction of vaginal gp120-specific IgA in mice following immunization with DNA prime and gp120 boost vaccine via either intranasal or intramuscular administrations [13]. Monkeys immunized with HIV gp41 antigens by both intramuscular and intranasal administrations were completely protected from intravaginal challenge with SHIV and elicited gp41-specific vaginal IgAs with HIV transcytosis-blocking properties and vaginal IgGs with neutralizing and/or antibody-dependent cellular-cytotoxicity (ADCC) activities [12]. It is apparent that the development and optimization of mucosal collection procedures to characterize antibodies induced by HIV vaccines at mucosal surfaces and establishment of correlates of mucosal protection are essential for evaluating vaccine candidates advancing to efficacy trials.

The following shortcomings in this study must be considered. First, participants had highly variable rest intervals from the last RV144 immunization to RV305 boosting which was much longer than ideal for a vaccine regimen to maintain antibody responses. Second, sample size, particularly for RS collections, was small. The RV306 trial (ClinicalTrials.gov NCT01931358) immunized Thai HIV-uninfected with RV144 regimen plus ALVAC-HIV/AIDSVAX^®^B/E or AIDSVAX^®^B/E boosts at week 48, or ALVAC-HIV/AIDSVAX^®^B/E at week 60 or 72. Humoral immune response assessments of the trial with bigger sample size of mucosal collection will help elucidate HIV vaccine-induced immune responses in mucosal compartments. Third, functional characterizations of antibody neutralization activity, binding affinity, avidity, and other effector functions such as ADCC, antibody-dependent cell-mediated virus inhibition (ADCVI), antibody-dependent cellular phagocytosis (ADCP) are not reported here and remain ongoing.

We showed that mucosal collection methodologies were successful in collecting and characterizing IgG and IgA immunoglobulins from anogenital secretions and that the RV144 regimen induced IgG antibodies to gp120 and gp70V1V2 scaffolds derived from CRF01_AE (92TH023) and subtype B (Case A2). Absence of IgA gp120 specific responses suggests that protection of mucosal surfaces in RV144 is likely IgG-mediated. The presence of HIV-specific IgG mucosal antibodies might have contributed to the modest protective effect against HIV acquisition in the RV144 trial in a population with mostly heterosexual transmission. Presence of IgG in CVM and rectal fluid could prevent HIV acquisition by several mechanisms but the protective role of IgG in seminal fluid is unclear. Immunoglobulins induced by HIV vaccines that are localized at the vascular epithelium of anogenital surfaces could be protective by sequestering and neutralizing viruses that penetrate the epithelial barrier. Collection and characterization of mucosal secretions would advance our understanding of the immune responses at mucosal surfaces to design and select efficacious HIV-1 vaccines in the future.
